# Does Water Temperature Affect the Timing and Duration of Remigial Moult in Sea Ducks? An Experimental Approach

**DOI:** 10.1371/journal.pone.0155253

**Published:** 2016-05-13

**Authors:** Anouck Viain, Magella Guillemette

**Affiliations:** Département de Biologie, Chimie et Géographie, Université du Québec à Rimouski, Rimouski, Quebec, Canada; Michigan State University, UNITED STATES

## Abstract

Aquatic birds have high cost of thermoregulation, especially during the moulting period, yet the effect of water temperature on the moulting strategy of aquatic birds has rarely been studied. Our general hypothesis is that energy savings associated with lower thermoregulation costs would be allocated to moulting processes. We predicted that aquatic birds moulting in warm water would have a higher level of body reserves, a faster growth rate of feathers, and an earlier remigial moult onset compared with birds moulting in cold water. We used the common eider (*Somateria mollissima dresseri*), a large sea duck, as the model species. Captive individuals were experimentally exposed to warm (18°C) and cold (8°C) water treatments during a three year period with individuals swapped between treatments. We found a similar feather growth rate for the two water temperature treatments and in contrast to our predictions, eiders exposed to warm water had a lower body mass and showed a delayed onset of remigial moult of approximately 7 days compared with those exposed to cold water. Our data indicate that body mass variations influence the timing of moult in unexpected ways and we suggest that it likely controls the occurrence of wing moult through a hormonal cascade. This study emphasizes the importance of improving our knowledge of the effects of water temperature on remigial moult of aquatic birds, to better assert the potential effects of global warming on their survival.

## Introduction

The timing of major events in the annual cycle of birds, such as reproduction and migration, has recently been the subject of several studies, especially in the context of global climate change. The timing of moult is an important factor that can affect the body condition of birds as well as their future survival [1,2]. Many living organisms adjust their activities and physiological functions according to the photoperiod, which is a reliable cue for the timing of seasonal events. Photoperiod is used to initiate functions that trigger migration, reproduction, and moulting in birds [3–5]. In addition to the endogenously determined annual cycles of birds, several studies have shown that birds can use secondary non-photoperiodic cues, such as ambient air temperature, food supply, and rainfall, to ‘fine tune’ their life cycle events to environmental variations [5–8].

Air temperature variations may have physiological consequences for moulting birds. Avian moult is associated with the disruption of body plumage, and for several species of birds, a higher thermal conductance has been observed during this period [9–13]. This effect is exacerbated in aquatic environments because water has a 23-fold higher thermal conductance, a 4-fold higher specific heat capacity, and a higher viscosity/density compared with air [14]. Consequently, the moult of aquatic birds may represent an important window for heat loss through water conduction and convection.

Feathers are inert integuments that have three main functions: body insulation, flight ability, and visual communication [15,16]. Throughout the year, feathers are damaged by mechanical abrasion induced by flight, reproduction, and foraging activities [17], and they are degraded by photochemical process or by parasitic and bacterial infections [18–20]. Thus, the maintenance of plumage integrity via the moult process is essential and one of the crucial events in the annual cycle of birds through which they replace worn feathers with new ones. During the prebasic moult, sea ducks drop all their flight feathers at once and become flightless [16,21,22] while they moult their body plumage, especially their belly feathers while remiges are growing [23] (A. Viain, *pers*. *obs*.). Because the synthesis of feathers entails widespread metabolic adjustments, requires a nutrient supply, and increases vascularization [10,12,24–28], moult is an energetically costly process, especially in synchronous flight-feather moulters, such as waterfowl species [29,30].

Dawson [31] and Visser *et al*. [32] showed an earlier initiation of prebasic moult at high temperatures for passerine birds. Barshep *et al*. [2] obtained similar results in male curlew sandpipers (*Calidris ferruginea*), although they found that the females started moulting earlier in years when the temperature in July was lower. Most waterfowl species undergo remigial moulting during summer or early autumn, which coincide with relatively warm water temperatures [33]. In a previous study, Guillemette and Butler [34] postulated that synchronising the remigial moult with periods of relatively warm water was energetically advantageous. In this study, we investigated the energy-conservation strategy hypothesis, which predicts that the energy conserved by lower thermoregulation costs in warm water will be re-allocated into higher body reserves, higher feather growth rate and an earlier remigial moult onset. Using Common Eiders (*S*. *m*. *dresseri*) as a model species, we applied an experimental approach where high and low temperature groups of individuals were observed and measured repeatedly during a three year period.

## Materials and Methods

### Ethics statement

This work was done under permit number SC-24 from Environment Canada following the principles of the *Guide to the Care and Use of Experimental Animal* and all manipulations were approved by the Canadian Council on Animal Care of the Université du Québec à Rimouski (CPA-44-11-96).

### Care of captive sea ducks

In 2010 and 2011, we collected Common Eiders eggs at Pointe-Métis in Québec, Canada from a breeding colony with an appropriate permit from Environment Canada. Eider ducklings (10 males and 7 females) were reared and imprinted on the experimenter for ease of handling. During the first 2.5 months, the handlers spent up to 10 hours per day with the ducklings training them to climb on a digital scale with a platform (Ohaus 5000 series T51P) and acclimating them to the measurements and manipulation. The ducklings were fed a Mazuri Waterfowl Starter Diet (#5641; 20% protein, 3.0% fat, and 6.5% fibre) until 3 months, and then a diet of two parts Mazuri Sea Duck Diet (#5681; 21.5% protein, 5.0% fat, and 4.5% fibre) to one part Mazuri Waterfowl Maintenance (#5642; 14.0% protein, 3.0% fat, and 5.0% fibre) *ad libitum* on a daily basis. The ducks were housed at Rimouski, Québec in an indoor enclosure (6.7 x 3.7 m in area) that supplied a natural photoperiod and contained a 6.0 x 1.7 m pool with continuously flowing fresh water at 0.4 m deep. Under these conditions, the eiders maintained seasonal cycles of moult in synchrony with the moults of free-living individuals (A. Viain, *pers*. *obs*.).

### Experimental design

The experiment was performed over 3 years (2011–2013). Our goal was to test the hypothesis that the energy conserved by lower thermoregulation costs in warm water would be advantageous for moulting eiders. From the end of June and prior to the moulting period, the indoor enclosure was separated into two parts. During the study period, the ambient temperature and the hygrometry were recorded daily, and the water temperature of the two swimming pools was recorded twice per day. The water temperature of the first swimming pool was maintained at 18°C (ranging between 17.4°C and 19.3°C for the warm-water treatment), and the second pool was maintained at 8°C (ranging between 7.0°C and 9.5°C for the cold-water treatment). The air temperature of both pools was maintained under 19°C (ranging between 10.0°C and 19.6°C) with an air conditioner, and the air humidity was maintained at 74% on average (ranging between 65% and 95%). Previous studies of thermoregulation in Common Eiders indicated that the lower critical temperature in air (LCT_AIR_) for the resting and winter acclimatized birds was 0°C (*n* = 7; [35]), whereas the LCT_AIR_ was higher in summer (7°C, *n* = 12 females; [36]). Jenssen *et al*. [35] evaluated an LCT of 15°C for winter acclimatized birds floating on the water (LCT_WATER_). Moreover, the lower oxygen consumption recorded in summer for eiders in air occurred between 7 and 21°C [36]. Consequently, the air temperatures used in our study (10–19°C) were well within the thermoneutral zone of moulting eiders. Previous studies have not evaluated the LCT_WATER_ value for eiders floating on the water in summer. However, the LCT_WATER_ in summer most likely exhibits a similar pattern as the LCT_AIR_ between the winter and the summer period, and we thus assume that the moulting birds exposed to cold water (8°C) were most likely under the LCT_WATER_, whereas the birds exposed to warm water (18°C) were within the thermoneutral zone. Therefore, under this assumption, eiders exposed to a cold water treatment would experience higher thermoregulation costs compared with birds exposed to warm water.

The eiders were separated into two groups: one group was placed in the warm-water treatment and the other group was placed in the cold-water treatment. The following year, the treatments were reversed. Thus, eiders hatched in 2010 (*n* = 10, 5 males and 5 females) underwent two moults in cold water and one in warm water, and those hatched in 2011 (*n* = 7, 5 males and 2 females) moulted once in warm water and once in cold water. During the 3 years of the experiment, 4 eiders died in winter (3 males and 1 female), and data from these 4 individuals were excluded from subsequent analyses (*n* = 13).

### Body mass and feather measurements

During the moulting period, the shedding dates of the 9^th^ primary (P9) the second from the outermost feather and 1^st^ secondary (S1) feather of each eider were noted as well as the emergence dates. Twice per week during feather growth, the lengths of the P9 and S1 feathers were measured with a Vernier calliper to the nearest 0.01 mm. The measurements were performed with the feathers flattened from the rim of the follicle to the distal tip of the feather, and the measurements repeated three times for both feathers. For each measurement, the time was noted for subsequent modelling of feather growth. All of the birds were weighed weekly (± 0.1 g), and the handling of each bird took approximately 2 to 4 min during the measuring session.

### Feather growth and flightlessness duration

We used the Gompertz model for modelling the feather growth of the P9 and S1 feathers, and for each bird and each feather, we estimated the *A*, *μ* and *λ* values as defined by the following function:

$$y(t)=A\cdot \text{exp}\left[-\text{exp}\left(\frac{\mu \cdot \text{exp}(1)}{A}\left(\lambda -t\right)+1\right)\right]$$

where *A* represents the asymptotic value, which is an estimate of the maximum length of the feather; *μ* represents the maximum feather growth rate; and *λ* represents the lag phase. We determined the growth rate of the P9 and S1 feathers to be between 5% and 90% of the final length of the feather by calculating the change in the feather length divided by the change in time. 5% growth in the model corresponds to a flight feather of few millimeters long which is the minimal length technically measured in the field. Finally, the duration of the flightless period of each moulting eider was calculated by determining the time between the shedding day of the old P9 feather (on growth curves corresponding to 1.5% of the P9 final length) and the day when the sea ducks could fly again, which was estimated at 88% of the final length of the P9 feathers. To determine this percentage, the eiders were trained to fly and respond to the call to take flight from the experimenter (the value was determined with 19 eiders, including 9 females and 10 males between 1 and 3 years of age; A. Viain, *unpublished data*).

### Thermal conductance

The estimation of thermal wet conductance was calculated using equation (1) of Jenssen *et al*. [35]: *C*_*w*_ = (*H* x *M*)/ ((*T*_*b*_*−T*_*a*_) x *A*), where *C*_*w*_ is in W.m^-2^.C^-1^; *H* represents the metabolic heat production (W.kg^-1^); *M* represents the body weight (kg); *T*_*b*_ represents the body temperature (°C), which was evaluated at 40.2°C [33]; *T*_*a*_ represents the ambient air or water temperature (°C); and *A* represents the surface area of the plumage (m^2^) and was estimated using the following formula: *A* = 0.097 x *M* ^2/3^. Jenssen *et al*. [35] indicated that the lowest heat production (*H*) of 3.83 W.kg^−1^ for winter-acclimatized eiders floating on the water was found for a water temperature between 16 and 25°C. Previous studies have shown a higher conductance associated with the moulting period of birds [9–13]. Consequently, it was assumed in this study that the value 3.83 W.kg^-1^ was the minimal metabolic heat production for moulting eiders exposed to 18°C water temperature. Under the LCT_WATER_, Jenssen *et al*. [35] indicated that the heat production of the winter-acclimatized eiders increased following the equation *H* (W.kg^-1^) = 5.48–0.09 *T*_*W*_ (*T*_*W*_ represents the water temperature in °C). Thus, in our experiment, we assumed that the minimal metabolic heat production for moulting eiders floating on cold water (8°C) was 4.76 W.kg^-1^.

### Analyses of time budgets

All of the activities were videotaped with a GoPro HERO3 throughout the flightless period. This monitoring was designed to determine the percentage of time that birds spent in and out of water during this period for the two water temperatures (cold and warm). The birds were checked daily to assess their moult stage, and all of the eiders (males and females) were in wing moult during the videotaped sessions. Each sampling session was recorded between 09:00 and 16:00 hours, and the number of birds in and out of the water was determined at every 5 min of the videotaped session. Approximately 940 min and 1300 min of video were analysed for the cold and the warm water temperatures, respectively.

### Statistical analysis

The data were analysed using the R platform (R 3.0.1. Development Core Team, 2013). To model the feather growth, we used the R package ‘*grofit*’ (v. 1.1, [37]). To model the effect of treatment and sex on the morphological and biometric variables, we ran linear mixed-effects models fitted with restricted maximum likelihood using the R package ‘*lme4*’ (v. 1.1–7, [38]) according to the treatment (warm or cold water) and sex as the fixed effects and bird identity as the random effect. In our experiment moult of one year old birds did not started earlier than moult of 2 and 3 year-olds. Thus we did not add the age term in our models. All of the variables were tested for homogeneity of variance and normality of distribution before proceeding with the parametric tests. We used Mann-Whitney tests (U) to evaluate changes in the mean percentage of time that the birds spent in and out of the water during the flightless period in the two water temperatures (cold and warm). All of the *P*-values were considered significant at the α = 0.05 level.

## Results

### Time budget of birds and thermal conductance

The birds spent approximately 26% of their time on the water and 74% of their time outside of the water regardless of the water temperature during the flightless period (27.5% and 72.5% (cold) and 24.2% and 75.8% (warm) in and out of the water, respectively; U = 11, *P* = 0.188).

Using the thermal wet conductance equation (1) provided by Jenssen *et al*. [35], the body temperature of moulting eiders (40.2°C; [33]) and the body mass at the beginning of moult in the cold and warm water were used to estimate the minimal thermal conductance (C), which was 1.9 W.m^-2^.°C^-1^ and 2.17 W.m^-2^.°C^-1^, respectively. Thus, the thermal conductance was 14% higher for individuals on warm water relative to individuals on cold water.

### Effect of temperature on body mass

At the beginning of the experiment, significant differences were not observed in the body mass between the two treatment groups and between sexes (Table 1). One week before the start of remigial moult and at the P9 shedding date, the eiders exposed to cold water were significantly heavier than those exposed to warm water for both sexes but markedly so in females (see statistical details in Table 1). In one month, the females exposed to cold water gained a mean of 307 g (16% of their initial body mass), whereas those exposed to warm water gained only 158 g (9%). The gain in body mass for males was less pronounced at 167 g (9%) for individuals exposed to cold water and 110 g (6%) for those exposed to warm water. At the end of remigial moult as well as one week later, the body mass of the eiders, especially the females, exposed to warm water was still lighter than the mass of those exposed to cold water, although the value was not significant (see statistical details in Table 1). One month after the end of the remigial moult, individuals exposed to cold water were significantly heavier than those exposed to warm water (Table 1). The body mass of the females exposed to cold water was 118 g higher (6.9%) on average than the mass of those exposed to warm water, whereas for males, the body mass was estimated as 122 g heavier (6.6%) for the cold-water treated birds.

**Table 1:** Dynamics of body mass during remigial moult of Common Eiders (Atlantic) (*Somateria mollisima dresseri*).

	Female mass (g, mean ± SE)		Male mass (g, mean ± SE)		Linear mixed-effects models		
	Cold	Warm	Cold	Warm	Treatment	Sex	Interaction
1 MB	1644.5 ± 53.7	1595.8 ± 43.1	1766.7 ± 28.9	1748.0 ± 37.4	F_1_,_19_ = 0.555 *p* = 0.465	F_1_,_11_ = 4.262 *p* = 0.062	F_1_,_19_ = 0.409 *p* = 0.530
1 WB	1896.2 ± 53.1	1696.3 ± 78.5	1882.8 ± 35.0	1821.5 ± 52.9	F_1_,_19_ = 19.658 *p* < 0.001	F_1_,_11_ = 0.522 *p* = 0.485	F_1_,_19_ = 6.007 *p* = 0.024
Start	1951.2 ± 45.3	1753.8 ± 66.5	1934.1 ± 32.6	1858.0 ± 48.1	F_1_,_19_ = 48.632 *p* < 0.001	F_1_,_11_ = 0.285 *p* = 0.604	F_1_,_19_ = 13.340 *p* = 0.002
End	1882.9 ± 50.5	1794.2 ± 70.5	1971.2 ± 49.7	1925.7 ± 56.0	F_1_,_19_ = 1.242 *p* = 0.279	F_1_,_11_ = 2.419 *p* = 0.147	F_1_,_19_ = 0.161 *p* = 0.693
1 WA	1903.7 ± 54.8	1777.2 ± 70.7	1953.2 ± 41.3	1943.9 ± 55.6	F_1_,_19_ = 1.514 *p* = 0.234	F_1_,_11_ = 2.288 *p* = 0.258	F_1_,_19_ = 1.360 *p* = 0.258
1 MA	1820.5 ± 39.9	1702.3 ± 40.9	1966.5 ± 45.5	1844.4 ± 44.2	F_1_,_19_ = 8.149 *p* = 0.010	F_1_,_11_ = 6.225 *p* = 0.030	F_1_,_19_ = 0.002 *p* = 0.967

Mean body mass (± SE) at six time points during the moult of female and male Common Eiders. 1 MB: one month before the shedding date; 1 WB: one week before the shedding date; start: at the shedding date of P9; end: at the end of the remigial moult; 1 WA: one week after the end of the remigial moult; and 1 MA: one month after the end of the remigial moult.

### Timing and duration of flight feather growth

Eiders from the experimental group exposed to warm water temperatures showed a delayed emergence date of both flight feathers P9 and S1 compared with birds in cold water, with a significant mean delay of 6.7 days and 7.3 days, respectively (see statistical details in Table 2). Treatment effects were not observed for the growth rates of P9 and S1 (Table 3). As a result, the flightlessness duration of eiders exposed to the two treatments did not differ (Table 2), with females presenting a flightlessness duration of 41.9 ± 1.7 days and males presenting a flightlessness duration of 43.7 ± 1.3 days.

**Table 2:** Flightlessness duration and emergence date of flight feathers of moulting Common Eiders (Atlantic) (*Somateria mollisima dresseri*) exposed to two water temperatures.

	Female (mean ± SE)		Male (mean ± SE)		Linear mixed-effects models		
	Cold	Warm	Cold	Warm	Treatment	Sex	Interaction
Emergence date of P9	16 August ± 3	26 August ± 3	12 August ± 4	16 August ± 4	F_1_,_19_ = 4.881 *p* = 0.041	F_1_,_11_ = 2.460 *p* = 0.145	F_1_,_19_ = 1.203 *p* = 0.286
Emergence date of S1	15 August ± 3	25 August ± 3	11 August ± 4	17 August ± 5	F_1_,_19_ = 5.020 *p* = 0.037	F_1_,_11_ = 1.218 *p* = 0.294	F_1_,_19_ = 0.813 *p* = 0.379
Flightlessness duration (days)	42.4 ± 0.3	41.0 ± 0.4	43.7 ± 0.6	43.7 ± 0.6	F_1_,_19_ = 2.241 *p* = 0.151	F_1_,_11_ = 10.457 *p* = 0.008	F_1_,_19_ = 2.249 *p* = 0.150

Mean emergence date of P9 and S1 (± SE) feathers and mean flightlessness duration (± SE) for male and female eiders exposed to two water temperatures (warm 18°C and cold 8°C). Significant differences were observed for the water treatments with respect to emergence date (P9, *p* = 0.041; S1, *p* = 0.037).

**Table 3:** Growth rate of flight feathers of moulting Common Eiders (Atlantic) (*Somateria mollisima dresseri*) exposed to two water temperatures.

	Female (mean ± SD)		Male (mean ± SD)		Linear mixed-effects models		
	Cold	Warm	Cold	Warm	Treatment	Sex	Interaction
P9 growth rate (mm.day^-1^)	3.94 ± 0.17	3.97 ± 0.23	3.84 ± 0.21	3.79 ± 0.18	F_1_,_19_ = 0.005 *p* = 0.946	F_1_,_11_ = 2.320 *p* = 0.155	F_1_,_19_ = 0.707 *p* = 0.411
P9 maximum growth rate (mm.day^-1^)	5.19 ± 0.21	5.23 ± 0.36	5.00 ± 0.29	4.95 ± 0.26	F_1_,_19_ = 2.5E-4 *p* = 0.988	F_1_,_11_ = 3.114 *p* = 0.104	F_1_,_19_ = 0.497 *p* = 0.489
S1 growth rate (mm.day^-1^)	3.55 ± 0.28	3.60 ± 0.19	3.39 ± 0.26	3.44 ± 0.18	F_1_,_19_ = 0.413 *p* = 0.528	F_1_,_11_ = 3.167 *p* = 0.101	F_1_,_19_ = 0.007 *p* = 0.935
S1 maximum growth rate (mm.day^-1^)	4.78 ± 0.38	4.79 ± 0.27	4.58 ± 0.34	4.61 ± 0.21	F_1_,_19_ = 0.087 *p* = 0.771	F_1_,_11_ = 2.366 *p* = 0.151	F_1_,_19_ = 0.018 *p* = 0.894

Mean growth rate of P9 and S1 feathers in mm.day^-1^ (± SD) and mean of the maximum growth rate of P9 and S1 in mm.day^-1^ (± SD) for male and female eiders exposed to two water temperature treatments (warm 18°C and cold 8°C). Significant differences were not observed among the water temperature treatments.

## Discussion

Few studies have quantified the direct effect of temperature on feather growth during the moulting process of birds. While previous studies have primarily evaluated the effect of air temperature on terrestrial species [31,32,39–42], in this study, we present the first investigation of the effect of water temperature on the remigial moult of a large diving duck using an experimental approach.

### Effect of water temperature on body mass during remigial moult

At the beginning of moult, the water temperature had an unexpected and significant effect on the body mass, and eiders exposed to 18°C water showed slower mass gains than those exposed to 8°C water. The thermal cost was assumed to be higher for moulting eiders exposed to cold water temperature because these temperatures are under the estimated range of their thermoneutral zone (see Methods); however, birds exposed to cold water may have compensated for the higher thermal costs by increasing their food intake beyond that of eiders exposed to the warm temperature treatment. Food was available *ad libitum*, and although we did not measure the food intake rate in this experiment, we observed that the rate of food disappearance was higher for the group exposed to cold water than for the group exposed to warm water (A. Viain, *pers*. *obs*.). Remigial moult imposes nutritional and energetic demands above those of general maintenance [16,29,30]. Indeed, moulting sea ducks increased their body mass at the beginning of wing moult, which then decreased it during the steep slope of feather growth, a period of high energy demand [22]. Portugal *et al*. [30] observed a similar pattern for moulting barnacle geese (*Branta leucopsis*) that were fed *ad libitum*, with the body mass peaking at the onset of wing moult and then dropping dramatically during moult. This suggests that this pattern to be general among waterfowl species. Thus, the amount of reserves that a bird can accumulate at the onset of moult might be beneficial and represents energy that can be reinvested in the process of feather replacement. The gain of mass is anticipatory, and this response was stronger for eiders exposed to the cold-water treatment. However, our results indicated an increase of 14% in the thermal wet conductance of eiders on warm water compared with that of eiders on cold water. This lower thermal wet conductance of individuals on cold water indicates greater isolation on cold water. Therefore, the gained mass at the beginning of moult might represent fat reserves that improve the isolation of eiders and reduce the thermal wet conductance.

### Effect of water temperature on the timing of moult and feather growth

Contradicting our energy-conservation strategy hypothesis, eiders moulting in warm water delayed the onset of remigial moult by 7 days compared to the cold-water treatment. This trend is in contrast with the data reported for four passerine birds where high air temperatures induced the onset of remigial moult whereas low temperatures inhibited it [31,32,39,40,43] but see [44] for an opposite trend. Shorebirds are known to start moult later in warmer tropical areas than the same species in colder temperature areas [21]. However, the mechanisms for this are still unknown. In our experiment we suggest the higher weight gain for eiders exposed to cold water might have caused the earlier onset of remigial moult because these birds accumulated larger body reserves. Czapulak [45] showed that the initiation of remigial moult in mute swans (*Cygnus olor*) was negatively correlated with their body condition, with males that had moulted later in the season presenting inferior body condition at moult onset. Jehl [46] observed that eared grebes (*Podiceps nigricollis*) must reach a threshold of fat accumulation (approximately 40 g) before remigial moult begins. We concur with these authors and hypothesized that moult initiation is modulated through hormonal mechanisms and body mass. For instance, thyroid gland activity peaks during moult [28,47–49], and the concentration of thyroid hormone circulating in the blood is related to temperature variations, high food intake and fat deposition [50–53].

Contrary to our predictions, eiders moulting in warm water did not show a higher rate of feather growth relative to birds exposed to cold water. Viain *et al*. [22] showed that little variance in feather growth rate occurred among various sea duck species, and this result is also supported by studies on birds in general [54–56]. Rohwer *et al*. [56] and Rohwer and Rohwer [57] suggested that an architectural constraint at the follicle level limits the feather growth rate of moulting birds. Such a constraint might explain the lack of differences in feather growth rate between the two water temperature regimes.

### Comparison with wild species

Viain [33] performed a literature review showing that most (84%) diving bird species moulting in the Northern Hemisphere did so during the period of the year where sea surface temperatures (SST) were highest. In addition, using data loggers that record flight occurrence and heart rate, Viain [33] showed that many individuals of the Baltic population of common eider (*S*. *m*. *mollissima*) moult in synchrony with the maximal SST, whereas those which did not experienced higher thermoregulation costs. Therefore, how can we reconcile the results of this study with those indicating that most diving birds moult in synchrony with peak SST? We suggest that one feature of our experimental approach might explain the discrepancy, is that food was given to the experimental individuals *ad libitum*. Food is patchily distributed in the wild [58,59], which requires to perform travelling flights to the feeding grounds and regular diving activity to the bottom [29,60]. Thus gaining body mass in the wild incurs extra feeding costs in contrast to captive birds eating pellet food. As a result, the strategy chosen by the cold-water individuals (gaining higher body mass) require little extra energy while it increases insulation. Another hypothesis that may explain this apparent difference is the adaptation of specific populations to regional temperature regimes. M. Guillemette (*unpublished data*) has found a strong correlation between daily body temperature and water temperature during a full annual cycle of *S*. *m*. *mollissima*, suggesting that this population is intimately adapted to its water temperature regime. Therefore, under this scenario, each subspecies of common eider could be adapted to local conditions, which would result in specific thermoneutral zone boundaries. Common Eiders have a circumpolar distribution, and the water temperatures they are exposed to during the moulting period are highly variable among the six subspecies (Table 4). The difference in the effects of water temperature on remigial moulting among the subspecies *S*. *m*. *dresseri* and *S*. *m*. *mollissima* may be the result of adaptive evolution among these two populations to their ‘colder’ or ‘warmer’ environments during remigial moult. Further investigations in a controlled environment would be required to test such a hypothesis.

**Table 4:** A comparative view of water temperatures encounter by six subspecies of Common Eiders (*Somateria mollissima* ssp.) during moult.

Species	SST (°C) during the moult period (between 2000 and 2014)*		Moulting area	References
	Mean	Range		
*S*. *m*. *borealis*	6.5	2.5–8.8	Gyrfalcon Archipelago, Ungava Bay, Disko Bay, Bell Inlet	[61,62]
*S*. *m*. *dresseri*	12.6	10.5–18.5	Southern and southwestern coastline of Anticosti island, north shore of the lower estuary (between Les Escoumins and Pointe à Boisvert), south shore of the lower estuary (between Cape Marteau and Matane), Pontbriand Bay, east of Baie-Johan-Beetz, coast of Maine and Nova Scotia	[63,64]
*S*. *m*. *faeroeencis*	12.4	11.6–13.4	Faeroe Island, northeast and west of Shetland	[65]
*S*. *m*. *mollissima*	17.5	15.6–21.1	Baltic Sea, Kattegat Sea, Wadden Sea	[66,67]
*S*. *m*. *sedentaria*	8.2	5.4–11.4	Hudson Bay, James Bay, western side of the Belcher Islands, Sleeper Islands	[68]
*S*. *m*. *v-nigrum*	7.0	3.3–10.6	Cape Parry, west side of Banks Island, north side of Prince Albert Sound, Cape Bathurst, south-central Coronation Gulf, Minto Inlet, Harrowby Bay, Kolyuchin Bay in Russia	[69]

Range and mean sea surface temperature (SST in °C) over 15 years (2000–2014) for the period between 15 July and 1 October in the moulting areas of six subspecies of common eider (*Somateria mollissima* ssp.).

* SST between 2000 and 2014 estimated from the database SST50 or SST14 of the National Oceanic and Atmospheric Administration (NOAA) http://www.class.ncdc.noaa.gov/saa/products/search?datatype_family=SST50 and http://www.class.ncdc.noaa.gov/saa/products/search?datatype_family=SST14.

In conclusion, our general hypothesis that energy savings associated with low thermoregulation costs would be allocated to moulting processes was not supported by our data. Nevertheless, we observed that water temperature can affect the timing of moult in diving bird species, and we suggest that the timing of remigial moult is modulated indirectly by the body condition of an individual. Finally, we propose that each population or subspecies of birds might be adapted to local moulting conditions. In the context of climate change, our observations highlight the importance of improving our knowledge of the effects of water temperature on the remigial moult of aquatic birds.
